# Case report: Detection and treatment of a rare submucosal gland duct adenoma of the esophagus

**DOI:** 10.3389/fonc.2024.1482068

**Published:** 2025-01-16

**Authors:** Yu Zhou, Daoxing He, Zhigang Huang, Zhaoyi Chen, Zheng Zhou, Hui Duan, Jie Xu

**Affiliations:** ^1^ Department of Gastroenterology, Xuancheng People’s Hospital, Anhui, China; ^2^ Electrocardiogram Room, Huai’an Third People’s Hospital, Jiangsu, China

**Keywords:** adenoma, esophageal submucosal gland duct, esophageal tumor, endoscopic resection, immunohistochemistry

## Abstract

This article reports a rare case of esophageal submucosal gland duct adenoma (ESGDA). The patient was found to have this tumor after undergoing endoscopy in an outpatient clinic due to occasional tingling while eating. White light endoscopy revealed the tumor as a dumbbell-shaped bulge, and ultrasound endoscopy revealed it as a hypoechoic mass located in the submucosal layer. The tumor was successfully resected by endoscopic mucosal dissection with no postoperative recurrence and a good prognosis. In conjunction with literature analysis, the diagnosis of ESGDA relies mainly on endoscopic and pathologic examinations, and its histologic features are highly specific. Despite the rarity of ESGDA, accurate histologic evaluation is essential to avoid misdiagnosis. Complete endoscopic resection is an effective treatment with a favorable long-term prognosis. Further studies remain necessary to reveal its pathogenesis.

## Introduction

Esophageal Submucosal Gland Duct Adenoma (ESGDA) is a rare benign esophageal tumor, with only 12 relevant cases reported according to a search of the PUBMED database, most of which were case reports. Having been given different names since it was first described in the 1990s (1), the pathologic features of ESGDA have not been fully standardized. This adenoma is usually found incidentally during endoscopy and patients are usually asymptomatic. On white light endoscopy, ESGDA lacks specific manifestations, and although several authors have described its ultrasonographic endoscopic features, immunohistochemical characteristics, and its ultrastructure (2, 3), however, ESGDA remains difficult to distinguish from other esophageal submucosal tumors, such as smooth muscle tumors and mesenchymal tumors.

From a broader perspective, tumors originating from the esophageal submucosal glands are themselves extremely uncommon, and ESGDA stands out as a particularly rare entity. Its distinctive double-layered epithelial architecture and lack of significant cellular atypia set it apart from more commonly encountered submucosal lesions. However, the low detection rate and nonspecific clinical presentation pose significant diagnostic challenges. Limited reports have suggested potential associations between ESGDA and other esophageal pathologies, such as Barrett’s esophagus or esophageal adenocarcinoma, though current evidence remains sparse and inconclusive. Further investigations are needed to elucidate the mechanisms underlying the development and possible malignant transformation of ESGDA, particularly the role of molecular factors like TP53. Such research is critical for advancing our understanding of this rare tumor’s pathology, improving diagnostic accuracy, and clarifying its potential oncogenic risk.

Although ESGDA usually presents as a benign lesion, its association with other esophageal diseases, but its potential association with other esophageal diseases, such as Barrett’s esophagus or esophageal adenocarcinoma, is unclear (3), therefore, the study of its pathogenesis and cancer pathway is of great clinical importance.

## Case report

**Figure 1:**
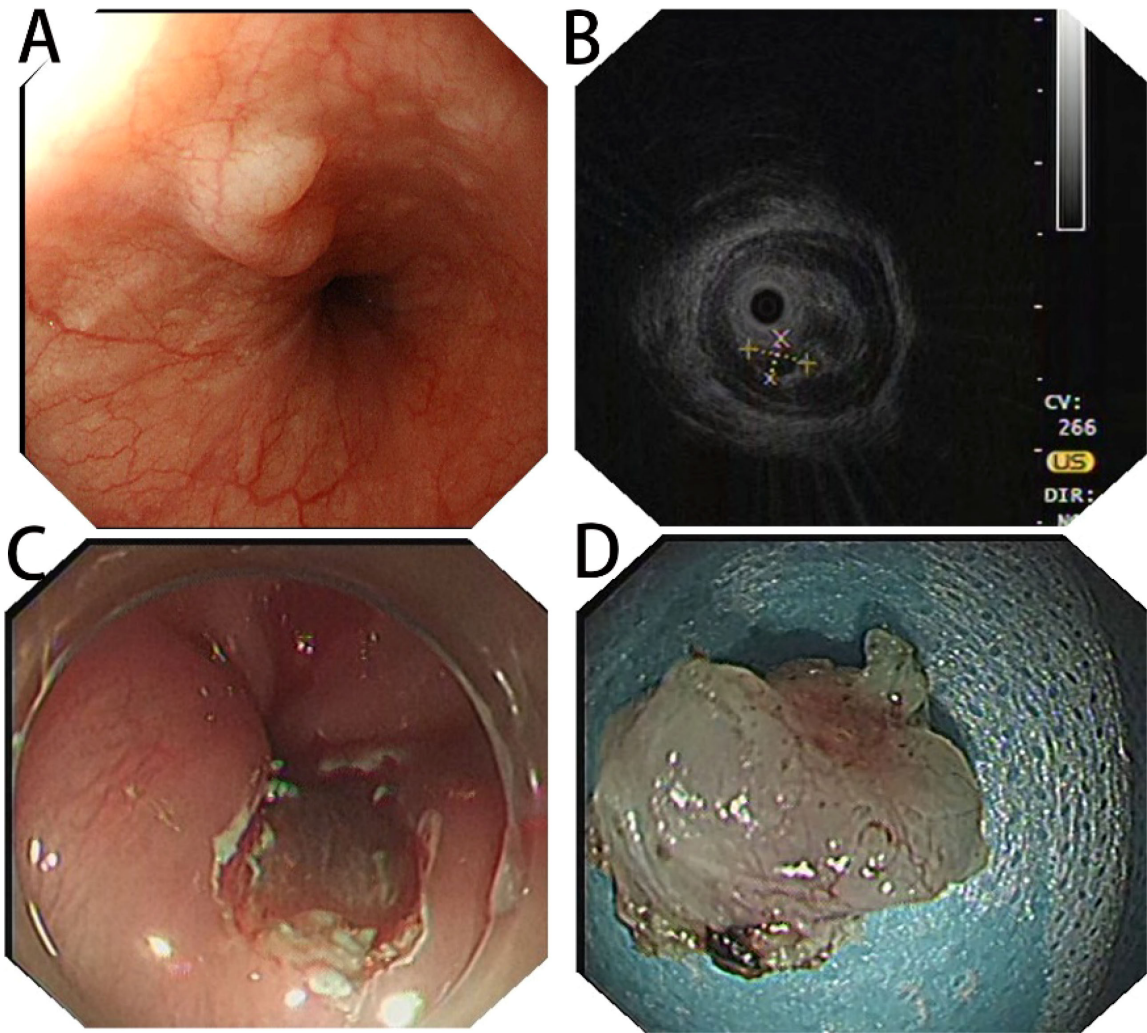
**(A)** White light endoscopy suggesting a dumbbell-shaped submucosal elevation in the lower esophagus; **(B)** preoperative EUS image showing a hypoechoic nodule in the submucosal layer with clear borders; **(C)** complete endoscopic resection of the lesion; **(D)** postoperative specimen.

**Figure 2:**
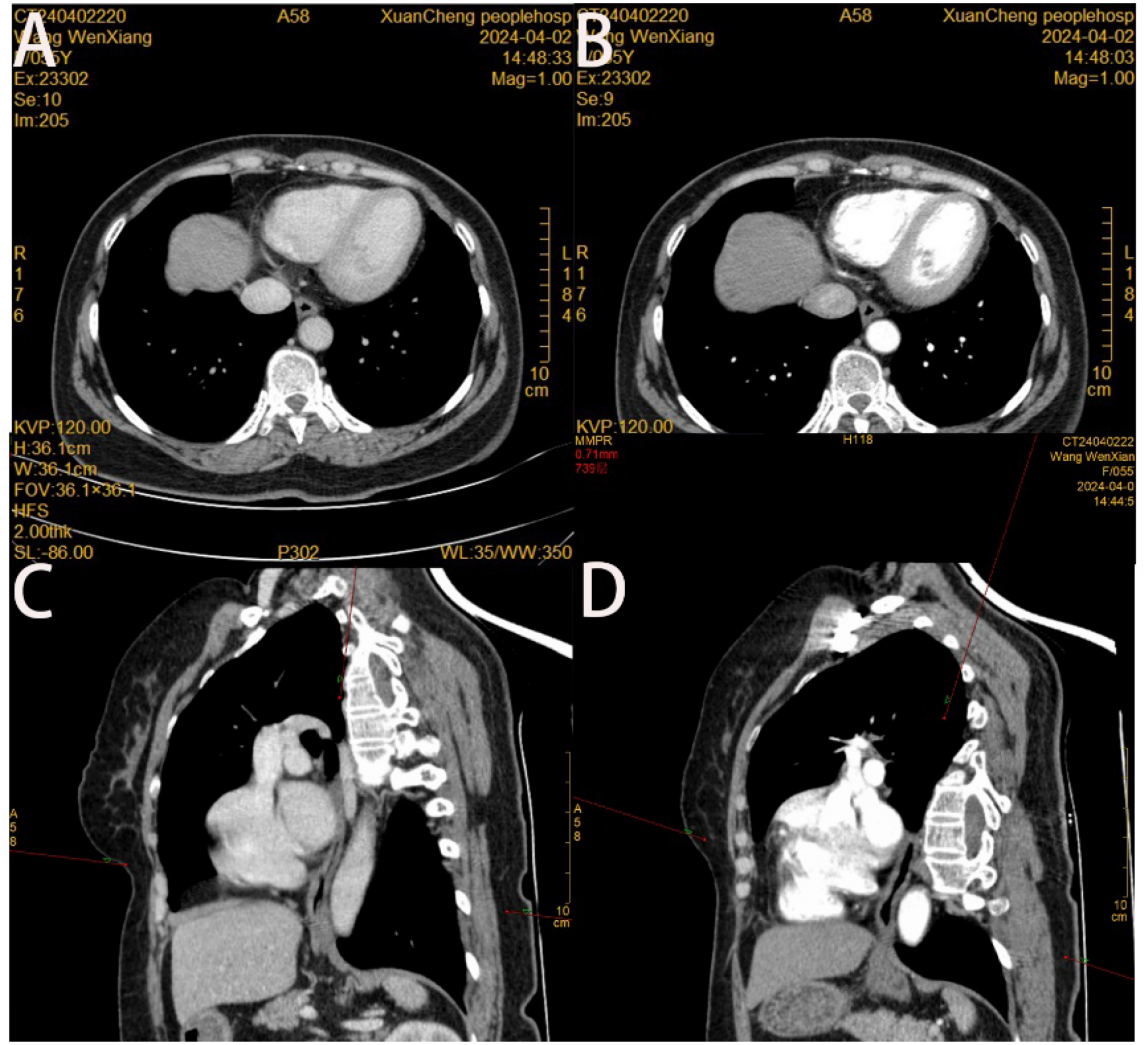
**(A)** Slight localized wall thickening of the lower esophagus is seen in the cross-sectional view of thoracic and abdominal enhanced CT in the venous phase; **(B)** No significant enhancement of the lower esophagus is seen in the cross-sectional view of thoracic and abdominal enhanced CT in the arterial phase; **(C, D)** no abnormality of the wall of the esophagus is seen in the oblique sagittal position of thoracic and abdominal enhanced CT in the venous phase and in the arterial phase.

Patient, female, 54 years old, had normal findings and no obvious abnormal signs. The patient underwent gastroscopy on March 19, 2024, due to a retrosternal tingling sensation after eating, which revealed a submucosal dumbbell-shaped bulge in the esophagus 33 cm from the incisors, approximately 0.6-0.8 cm in size (**Figure 1A**). The patient was hospitalized in the department of gastroenterology on April 1, 2024, for further diagnosis and treatment. The patient had a history of cholecystectomy, no family history of genetic or chronic diseases, and no history of smoking or alcohol consumption. Complete Blood Count (CBC): Hemoglobin (Hb) 125 g/L, White Blood Cell count (WBC) 4.94 × 10⁹/L, Platelet count (PLT) 305 × 10⁹/L, Neutrophil count 2.74 × 10⁹/L, Lymphocyte count 1.83 × 10⁹/L; all other parameters were within normal limits. Biochemistry: Total Bilirubin (TBIL) 39.9 µmol/L, Direct Bilirubin (DBIL) 8.1 µmol/L, Indirect Bilirubin (IBIL) 31.8 µmol/L, Alanine Aminotransferase (ALT) 23.1 U/L, Aspartate Aminotransferase (AST) 14.7 U/L, Alkaline Phosphatase (AKP) 103.6 U/L, Gamma-Glutamyl Transferase (GGT) 16.5 U/L, Creatinine (Cr) 36.2 µmol/L, Glucose (GLU) 5.51 mmol/L, Cholesterol (CHOL) 5.25 mmol/L, Triglycerides (TG) 1.75 mmol/L; all other parameters were within normal limits. Coagulation Function: Prothrombin Time (PT) 11.0 s, International Normalized Ratio (INR) 0.90, Prothrombin Time percentage (PT%) 129.40%, Activated Partial Thromboplastin Time (APTT) 35.6 s, Fibrinogen (FIB) 2.69 g/L. Immunological Markers: HBsAg, HBsAb, HBeAg, HBeAb, HBcAb, HCV-Ab, and HIV-Ab were all negative. Tumor Markers: Alpha-Fetoprotein (AFP) 2.17 µg/L, Carcinoembryonic Antigen (CEA) 2.35 µg/L, Cancer Antigen 125 (CA125) 7.20 µg/L, Cancer Antigen 19-9 (CA19-9) 5.85 µg/L. Electrocardiogram suggested sinus rhythm with T-wave changes. Chest contrast-enhanced CT revealed localized wall thickening of the lower segment of the esophagus without significant enhancing lesions, with clear surrounding fat interstices. Additionally, small nodular lesions were identified in the lower lobes of both lungs, and small gallstones were present in the gallbladder. (**Figure 2**). Hepatobiliary and urologic ultrasound suggested fatty liver and sand-like stones in both kidneys.

**Figure 3:**
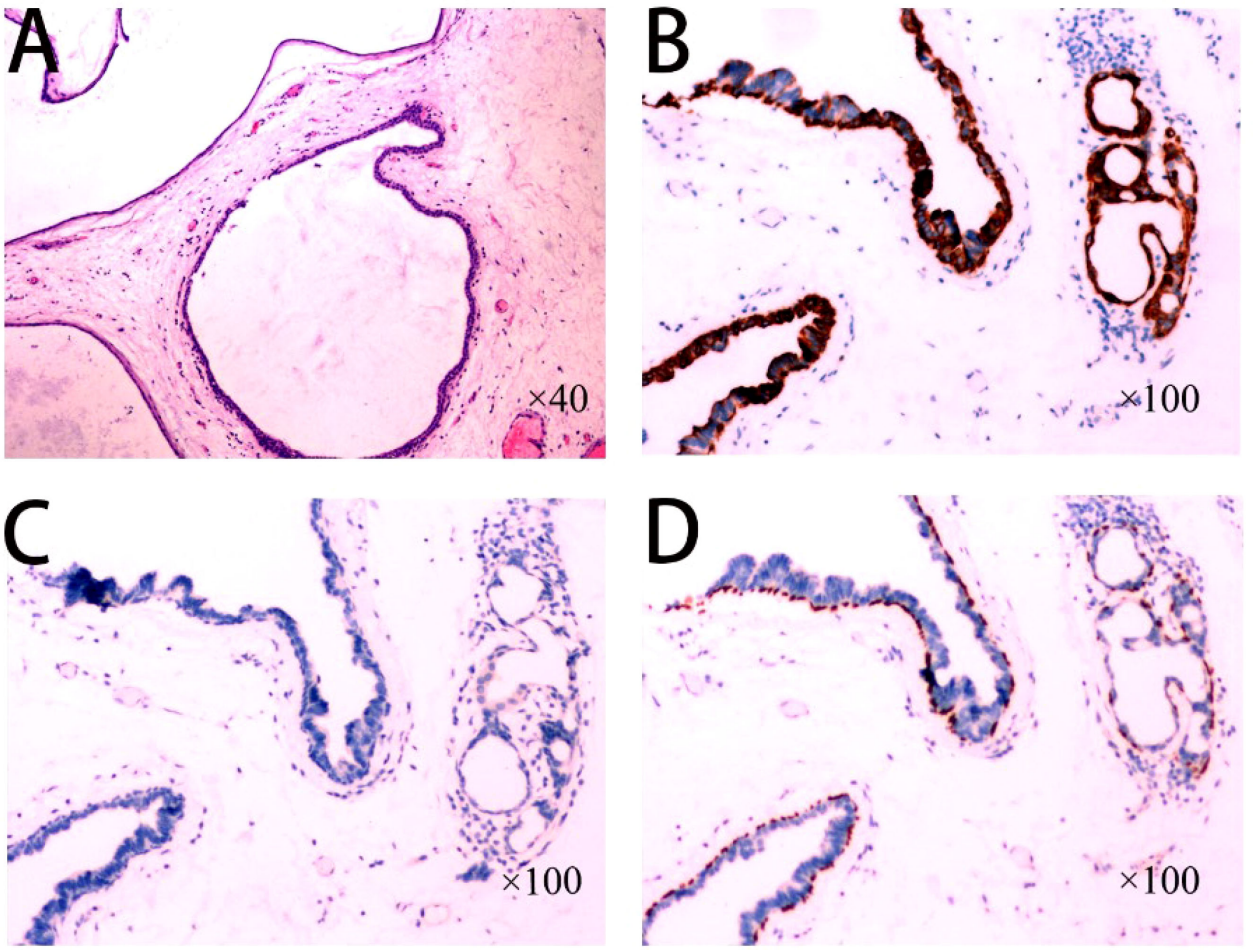
**(A)** High-magnification view of the tumor consisting of multiple dilated ducts or cysts arranged in a gland duct-like pattern, with medial gland epithelial cells and lateral basal cells visible in the dilated ducts; **(B)** Immunohistochemistry showing CK5/6 (basal cells +); **(C)** Immunohistochemistry MUC2 (-), MUC5AC (-), MUCS6 (-); **(D)** Immunohistochemistry P63 (basal cells +).

Treatment: Preoperative ultrasonographic endoscopy suggested a hypoechoic esophageal region 33 cm from the incisors, with a lesion originating in the submucosal layer (**Figure 1B**), measuring approximately 0.6 cm in size and of undetermined nature. The patient underwent endoscopic mucosal dissection under endotracheal intubation anesthesia (**Figure 1C**) with complete resection of the tumor (**Figure 1D**). Postoperative pathological findings confirmed the diagnosis of esophageal submucosal gland duct adenoma (**Figure 3A**). Immunohistochemical analysis showed CK5/6 (basal cell +) (**Figure 3B**), MUC2 (-), MUC5AC (-), MUCS6 (-) (**Figure 3C**), and P63 (basal cell +) (**Figure 3D**).

## Discussion

Esophageal submucosal gland duct adenoma (ESGDA) is a very rare benign tumor of the esophagus, usually detected incidentally during endoscopic examination and mostly asymptomatic, though some patients may present with dysphagia (4). Diagnosis relies primarily on endoscopic and pathological evaluation. Endoscopically, ESGDA typically appears as a hemispherical or dome-shaped elevation with a smooth, broad-based surface. Endoscopic ultrasound generally shows a hypoechoic lesion in the muscular to submucosal layer, sometimes with cystic components (5). It commonly occurs in the lower esophagus and may be associated with long-term gastroesophageal reflux disease (GERD) (6). A limited number of case reports suggest a potential association with Barrett’s esophagus or esophageal adenocarcinoma, though further studies are warranted (3). Most reported cases are single case reports or small case series, highlighting the rarity of this disease and underscoring the importance of accurate histological evaluation (7, 8). According to the diagnostic criteria proposed by Nie et al. (4), ESGDA features multiple ductal cysts lined with a dual layer of epithelial cells, duct epithelial hyperplasia, possible gland follicular atrophy, and lymphocytic infiltration. Immunohistochemically, the inner epithelial layer is positive for CK7 and CK8/18, while the basal layer cells are p63 and CK5/6 positive, and cells are negative for CK20, CDX-2, MUC2, MUC5AC, and MUC6 (3). Although rare, ESGDA can be confused with other pathologies, particularly adenocarcinoma, but its distinct double-layered epithelium lacking cellular heterogeneity and nuclear atypia is key to differentiation (3). Additionally, ESGDA is predominantly located in the lower esophagus and must be differentiated from benign epithelial tumors of the gastroesophageal junction, such as gastric adenomas. Under endoscopy, gastric adenomas also present as polypoid elevations and can be histologically classified into four types: intestinal type, small depressed type, pyloric type, and acid-secreting type. Intestinal type adenomas exhibit glandular structures with surrounding stromal inflammatory cell infiltration, primarily located in the distal stomach, and are composed mainly of atypically proliferating intestinalized epithelial cells. Small depressed type adenomas and ESGDA both show cystic dilatation; however, small depressed type adenomas are characterized by typical mucous caps and positive staining for mucin 5AC and PSA. The pyloric and acid-secreting types of adenomas have eosinophilic cytoplasm, similar to ESGDA, but the former two types feature glandular structures that align in cord-like patterns, forming irregularly branching tubules with scattered parietal cells and varying degrees of mucin expression, possessing the potential to develop into adenocarcinoma. These distinguishing features necessitate a comprehensive evaluation combining clinical presentation, histological morphology, and immunophenotype to ensure accurate diagnosis (9, 10).

Due to its benign nature, endoscopic resection is considered the primary and curative treatment, with excellent prognosis and no reported recurrence or malignant transformation (11). In recent years, the application of robotic-assisted surgical techniques in the field of esophageal surgery has emerged as a significant and innovative frontier. Evidence suggests that completely robotic-assisted Ivor Lewis esophagectomy combined with intrathoracic robotic-sewn anastomosis is technically feasible, safe, and comparable to conventional methods in terms of perioperative complications, oncological outcomes, and long-term survival (12). Although endoscopic resection remains effective for ESGDA, future integration of robotic-assisted approaches may offer enhanced precision and potentially broaden treatment options for rare and anatomically challenging esophageal lesions. Information regarding the pathogenesis of ESGDA remains limited, and some studies implicate TP53 as a potential oncogene (13, 14). TP53, as a crucial tumor suppressor gene, prevents carcinogenesis by repairing DNA damage and regulating the cell cycle. Mutations in TP53 are considered early events in the carcinogenic process of various esophageal tumors. Studies have shown that TP53 mutations play significant roles not only in malignant tumors but also in certain benign tumors. In the context of ESGDA, TP53 mutations may be associated with its atypical biological behavior. Although ESGDA presents clinically as a benign lesion, the presence of TP53 mutations in some cases may indicate a potential risk of malignant transformation or at least provide a molecular basis for excessive cellular proliferation and tumor formation. Additionally, TP53 mutations leading to the loss of tumor suppressor functions and genomic instability may be key molecular mechanisms in the development of ESGDA. These findings highlight the importance of further investigating the specific mechanisms of TP53 mutations in ESGDA, offering valuable directions for future diagnostic and therapeutic strategies.

## Conclusions

Esophageal submucosal gland duct adenoma (ESGDA) is a rare, benign esophageal tumor characterized by distinct histologic features and generally detected incidentally. Complete endoscopic resection remains the primary treatment, offering excellent long-term outcomes and minimal risk of recurrence. Although the role of TP53 in its malignant transformation has been suggested, current evidence is limited, warranting further investigation into the molecular mechanisms underlying ESGDA. Moreover, as innovative surgical techniques continue to evolve, recent findings in the field of robotic-assisted esophageal surgery demonstrate the feasibility, safety, and efficacy of such advanced approaches. While endoscopic resection is effective for treating ESGDA, the integration of robotic-assisted techniques could broaden future therapeutic options for more complex or challenging lesions, ultimately improving patient care and expanding the horizons of minimally invasive esophageal surgery.

## Data Availability

The original contributions presented in the study are included in the article/supplementary material. Further inquiries can be directed to the corresponding author.

## References

[1] Tsutsumi M Mizumoto K Tsujiuchi T Maruyama H Koizumi M Inagaki T . Serous cystadenoma of the esophagus. Acta Pathol Jpn. (1990) 40:153–5. doi: 10.1111/j.1440-1827.1990.tb01555.x 2339602

[2] Agawa H Matsushita M Kusumi F Nishio A Takakuwa H . Esophageal submucosal gland duct adenoma: characteristic EUS and histopathologic features. Gastrointest Endosc. (2003) 57:983–5. doi: 10.1016/s0016-5107(03)70058-3 12776064

[3] Harada O Ota H Katsuyama T Hidaka E Ishizaka K Nakayama J . Esophageal gland duct adenoma: immunohistochemical comparison with the normal esophageal gland and ultrastractural analysis. Am J Surg Pathol. (2007) 31:469–75. doi: 10.1097/01.pas.0000213400.64945.9e 17325490

[4] Nie L Li W Xue L Wang L Shen Y Fan X . Submucosal gland neoplasms of the esophagus: an update and review. Esophagus. (2020) 17:376–84. doi: 10.1007/s10388-020-00758-1 PMC7497321 32621256

[5] Matsushita M Okazaki K . Esophageal, submucosal, gland duct adenoma: role of EUS for endoscopic removal. Gastrointest Endosc. (2005) 61:790–1. doi: 10.1016/s0016-5107(05)00142-2 15856000

[6] Takubo K Esaki Y Watanabe A Umehara M Sasajima K . Adenoma accompanied by superficial squamous cell carcinoma of the esophagus. Cancer. (1993) 71:2435 –2438. doi: 10.1002/1097-0142(19930415)71:8<2435:aid-cncr2820710802>3.0.co;2-a 8453568

[7] Hua HJ Yang QY Li KD Li H . Zhonghua bing li xue za zhi. Clinicopathological characteristics of esophageal submucosal gland duct adenoma (2021) 50:1266–8. doi: 10.3760/cma.j.cn112151-20210330-00247 34719167

[8] Genere JR Lee HE Wu TT Prichard DO Wang KK . Endoscopic findings of esophageal submucosal gland duct adenoma. VideoGIE. (2019) 4:361–3. doi: 10.1016/j.vgie.2019.04.001 PMC6669342 31388611

[9] Shibagaki K Mishiro T Fukuyama C Takahashi Y Itawaki A Nonomura S . Sporadic foveolar-type gastric adenoma with a raspberry-like appearance in Helicobacter pylori–naïve patients. Virchows Archiv. (2021) 479:687–95. doi: 10.1007/s00428-021-03124-3 34043063

[10] Ueyama H Yao T Akazawa Y Hayashi T Kurahara K Oshiro Y . Gastric epithelial neoplasm of fundic-gland mucosa lineage: proposal for a new classification in association with gastric adenocarcinoma of fundic-gland type. J Gastroenterol. (2021) 56:814–28. doi: 10.1007/s00535-021-01813-z PMC8370942 34268625

[11] Nie L Wu HY Shen YH Fan XS Sun Q Huang Q . Esophageal submucosal gland duct adenoma: a clinicopathological and immunohistochemical study with a review of the literature. Dis Esophagus. (2016) 29:1048–53. doi: 10.1111/dote.12442 26542981

[12] Huscher CGS Cobellis F Lazzarin G . Intrathoracic robotic-sewn anastomosis during ivor lewis esophagectomy for cancer: back to basics? J Gastrointest Surg. (2023) 27:1034–41. doi: 10.1007/s11605-023-05616-w 36732403

[13] Dees ND Zhang Q Kandoth C Wendl MC Schierding W Koboldt DC . MuSiC: identifying mutational significance in cancer genomes. Genome Res. (2012) 22:1589–98. doi: 10.1101/gr.134635.111 PMC3409272 22759861

[14] Zhu Z Qin B Wang B Wang Z . Whole-exome sequencing of esophageal submucosal gland duct adenocarcinoma. Nat Genet (2021) 45(5):478–86. doi: 10.21203/rs.3.rs-791813/v1 PMC3678719 23525077

